# Impact of MET alterations on targeted therapy with EGFR-tyrosine kinase inhibitors for EGFR-mutant lung cancer

**DOI:** 10.1186/s40364-019-0179-6

**Published:** 2019-11-21

**Authors:** Zhe Zhang, Sen Yang, Qiming Wang

**Affiliations:** Department of Internal Medicine, The Affiliated Cancer Hospital of Zhengzhou University, Henan Cancer Hospital, Zhengzhou, 450008 China

**Keywords:** MET amplification, EGFR-TKIs, Resistance, Non-small cell lung cancer

## Abstract

EGFR-tyrosine kinase inhibitors (EGFR-TKIs) have achieved remarkable outcomes in the treatment of patients with EGFR-mutant non-small-cell lung cancer, but acquired resistance is still the main factor restricting their long-term use. In addition to the T790 M mutation of EGFR, amplification of the MET (or c-MET) gene has long been recognized as an important resistance mechanism for first- or second-generation EGFR-TKIs. Recent studies suggest that a key mechanism of acquired resistance to third-generation EGFR-TKIs (such as osimertinib) may be MET amplification and/or protein overactivation, especially when they are used as a first-line treatment. Therefore, in patients resistant to first-generation EGFR-TKIs caused by MET amplification and/or protein overactivation, the combination of osimertinib with MET or MEK inhibitors may be considered.

## Introduction

Lung cancer is the leading cause of cancer-related death in humans, accounting for approximately one-third of cancer-related deaths worldwide. Non-small-cell lung cancer (NSCLC) is the main type and accounts for more than 80% of lung cancer cases, with an overall 5-year survival rate of approximately 18% [1]. In the past few decades, great efforts have been made to treat lung cancer worldwide, but the survival rate has not been significantly improved. The discovery of EGFR mutations and the advent of EGFR-tyrosine kinase inhibitors (EGFR-TKIs) for the treatment of metastatic NSCLC has dramatically changed the prognosis of selected patients and become an important milestone in NSCLC targeted therapy.

The proportion of EGFR mutations varies from race to race and is not the same in Western and Asian NSCLC populations, in which it is approximately 15 and 40%, respectively [2]. EGFR mutations mainly occur in four exons (exons 18–21), and the most common mutations are exon 19 deletions (approximately 60%) and exon 21 L8585R point mutations (approximately 30%), accounting for approximately 90% of all EGFR mutations [2]. EGFR mutations primarily increase the affinity between EGFR-TKIs and mutant receptors and are therefore sensitive to EGFR-TKIs. The first generation of EGFR-TKIs, such as gefitinib and erlotinib, blocks the further transmission of signals into cells by competitively binding to ATP-binding EGFR tyrosinase catalytic domain binding sites on the cell surface, thus inhibiting tumor cell growth and inducing apoptosis. Treatment of NSCLC harboring EGFR mutations with first generation of EGFR-TKIs is widely used in the clinic has achieved great success [3]. Unfortunately, patients eventually develop acquired resistance leading to disease progression, which is also why the long-term application of these drugs is limited [2, 4, 5].

Approximately 60% of acquired resistance to the first generation of EGFR-TKIs results from EGFR exon 20 T790 M mutations. In addition, several studies have found that amplification of the MET (also referred to as c-MET) gene is also an acquired resistance mechanism that leads to the failure of EGFR-TKI treatment [6]. The data show that MET gene amplification is present in approximately 5–22% of patients with NSCLC who develop acquired resistance to the first generation of EGFR-TKIs [2, 4, 5]. There are also studies illustrate that Met expression and activation (before EGFR TKI treatment) cause poor response to subsequent EGFR inhibitor treatment, despite the presence of EGFR TKI sensitizing mutations, this part of the patient is rare [6, 7]. MET bypasses the suppressed EGFR phosphorylation kinase pathway and is amplified through the ERBB3-P13K/AKT and MAPK-ERK1/2 T pathways. Amplified c-MET promotes downstream signal transduction through bypass activation to avoid cell death by EGFR-TKIs. This promotes the proliferation of cancer cells, which ultimately leads to the resistance of patients to EGFR-TKIs. Therefore, it is necessary to simultaneously inhibit EGFR and MET to overcome the EGFR-TKI resistance caused by MET amplification [8, 9]. Although MET amplification can occur with the T790 M mutation, approximately 60% of MET amplifications do not involve the T790 M mutation. There is a negative correlation between T790 M and MET amplification, indicating that these two mechanisms have complementary or independent roles in acquired resistance [10].

Osimertinib (AZD9291 or TAGRISSO™) is representative of the third generation of EGFR-TKIs and has been approved by the FDA for patients with locally advanced NSCLC or NSCLC patients who are positive for the EGFR T790 M mutation. Currently, it is also approved as the first-line treatment for patients with NSCLC harboring EGFR mutations (exon 19 deletion or exon 21 L858R mutation). Although osimertinib has achieved great clinical success, it still cannot avoid acquired resistance. Apart from some of the mechanisms involved in C797S mutations and MET amplification, the mechanism of resistance is largely unknown [11]. For the C797S mutation, a fourth generation of EGFR-TKIs, such as EAI045, has been developed [12]. This review will focus on the role of MET amplification in the acquired resistance of osimertinib and other third-generation EGFR-TKIs.

## MET structure and function

**Fig. 1:**
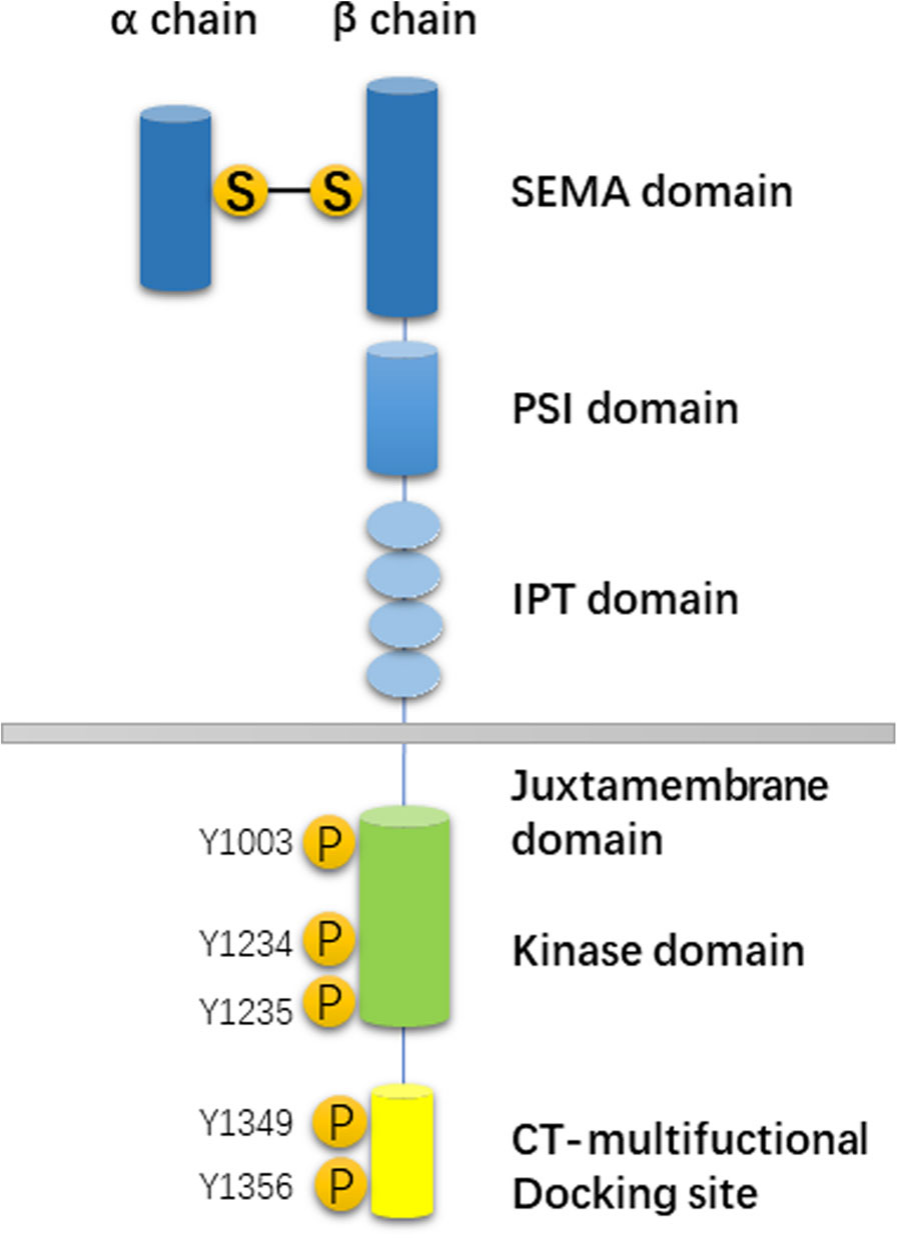
Protein structure of MET

MET is a proto-oncogene located in the long arm of human chromosome 7 (7q21–31); it is approximately 125 kb in length and contains 21 exons [13]. Its protein product c-MET is a tyrosine kinase receptor, which contains structural regions such as the Sema region and 4 IPT regions, including the PSI region, JM region, TK region and TM region. The Sema region is a ligand-binding region, and the JM region contains several tyrosine phosphorylation sites and has a function of initiating tyrosine kinase activity [13, 14]. The ligand of MET is human hepatocyte growth factor (HGF), belonging to the plasminogen family, that consists of the N-terminal, Kringle domain and C-terminus. Mature HGF is a heterodimer formed by a disulfide bond between the α chain and β chain produced by a proteolytic enzyme in a precursor and has the function of activating MET [3, 15]. When HGF binds to MET, the autophosphorylation of Y1234 and Y1235 in the intracellular tyrosine kinase domain occurs, resulting in the autophosphorylation of Y1349 and Y1356 at the C-terminal multifunctional docking site (Fig. 1). This induces the recruitment of several intracellular effector adaptor proteins, such as growth factor receptor binding protein 2 (Grb2), GAB1, SRC and PI3K, thereby activating downstream signaling pathways [14, 16]. The HGF/MET signaling pathway is expressed in both embryonic and adult bodies under certain physiological conditions [17]. During embryonic development, the HGF/MET signaling pathway plays an important role in promoting mitosis and inducing morphogenesis; in adults, this signaling pathway plays a role in repair and regeneration after tissue damage. The main types of HGF/MET signaling pathway variants in NSCLC patients are a few mutations, amplifications, exon 14 skip mutations and rearrangements [15, 18].

The role of MET amplification and protein hyperactivation in conferring resistance to third-generation EGFR-TKIs was assessed. We found that the EGFR mutation NSCLC cell line (HCC827/ER) resistant to erlotinib had MET gene amplification and protein overactivation and was cross-resistant to osimertinib and rociletinib. In addition, HCC827 cells (HCC827/AR), which have acquired resistance to osimertinib, also show MET gene amplification and protein overactivation. Compared with the parental cell line, the p-MET level is increased, and these cells are not only resistant to rociletinib but also to erlotinib [19]. With small molecule MET inhibitors or knockdown of MET gene expression, osimertinib restores the ability to inhibit HCC827/ER and HCC827/AR cell growth and inactivate ErbB3 or inhibit ErbB3 phosphorylation in vitro and in vivo. Our studies suggest that MET amplification and protein overactivation may be a common resistance mechanism for the first and third generation of EGFR-TKIs. Treatment with osimertinib or other third-generation EGFR-TKI monotherapy may be ineffective due to MET amplification and/or protein overactivation.

Recent studies have reported similar results. Many laboratories constructed HCC827 cell lines resistant to erlotinib with MET amplification and protein overactivation, and the cells were resistant to osimertinib [20]. H1975-P1 cells resistant to AC0010 were derived from a nude mouse model of H1975 xenograft tumors in nude mice. After 3 months of treatment or selection with AC0010, the cells overexpressed the MET gene, MET protein and p-MET gene and were resistant to afatinib, osimertinib and rociletinib [21]. In the PC-9/NaqR2 cell line (derived from the EGFR mPC-9 cell line), MET amplification was also detected with elevated levels of MET and p-MET. This cell line is resistant to gefitinib but sensitive to the combination of naquotinib and MET inhibitors (crizotinib or SGX532).

## Detection of MET dysregulation

Dysregulated MET expression and activity in human cancer tissues can be detected at the gene and protein levels. The following assays were performed to determine MET dysregulation. Fluorescence in situ hybridization (FISH): the MET gene copy numbers were obtained by detecting the site number of MET and CEP7 (as the control). Its advantage is high accuracy, good repeatability, good correlation with the curative effect, and use of fewer specimens; however, MET protein expression on the cell surface but not MET amplification could be detected [22]. Droplet digital PCR (ddPCR): the difference in fluorescence signal strength between the amplificated MET site and internal reference site was detected. Its advantages are high accuracy and rapid detection speed, but a large amount of high-quality DNA fragments is required [23]. Immunohistochemistry (IHC): tissues and cells positive for MET expression were identified by evaluating the staining status of the cells. Based on MetMAb criteria, a staining score of 2 or 3 is defined as high MET expression, whereas a score of 0 or 1 is defined as low MET expression. The advantages of IHC are that it is a vetted technique, produced rapid and repeatable results in many cases, allows for simultaneous observation of cell morphology, and has a low cost, but the disadvantages are the subjective interpretation of the results ease of sample disruption during the testing process [24]. Next-generation sequencing (NGS): copy number variation (CNV) can be estimated by calculating the sequencing depth of the region where the MET gene is located. Its advantages are the parallel detection of multiple genes from a single tissue or blood sample, the detection of all mutations present at one time, and its high sensitivity. Its shortcomings are high cost, requirement of specialized NGS sequencing equipment, and extensive technical requirements to perform the assay [25].

A recent study showed that liquid biopsy to detect MET is feasible. MET changes occurred in 7.1% of patients on liquid biopsy, which was higher than the frequency found in tissue (1.14%; *P* = 0.0002). The study included ctDNA sequencing results from 438 patients and analyzed the relationship between MET expression and clinicopathological parameters. The results showed that MET ctDNA alterations were associated with a poorer prognosis and higher numbers of genomic abnormalities and bone metastases [26].

## MET amplification

An increase in the copy number of the MET gene can occur in either the polyploid or amplification process. Polyploidy is the replication of a chromosome, and amplification is the replication of a local or regional gene. FISH can be used to detect the value of MET/CEP7 to distinguish between polyploidy and amplification. Polyploidy does not involve driver genes, and MET amplification may be a driver gene mutation and one of the main mechanisms of EGFR-TKI resistance. There is no consensus on how many copies of MET are defined as positive. One study used FISH to evaluate MET copy number in lung adenocarcinoma, associated clinical and molecular characteristics were captured. The MET/CEP7 ratio is currently classified into low (< 2.2), medium (> 2.2, < 5) or high (≥ 5), A MET/CEP7 FISH ratio of 5 or higher is defined a “MET-positive” group with no oncogenic overlap. As this method and criteria are also associated with the highest response rate to MET inhibition, they represents the clearest definition of a MET copy number gain-addicted state [27, 28].

## MET overexpression

MET overexpression can be caused by gene amplification, gene mutation, transcriptional enhancement, or posttranscriptional mechanisms. Immunohistochemistry (IHC) can be used to detect the overexpression of MET in tissue specimens. In different studies, the proportion of MET overexpressed in NSCLC varies greatly from 15 to 70% due to differences in experimental reagents and threshold settings.

## Detection of MET amplification

MET amplification was detected in third-generation EGFR-TKI-resistant EGFR-mutant NSCLC, but the results from different studies were not identical. Le et al. [20] detected 5 MET amplifications in 42 patients with osimertinib resistance, accounting for approximately 14%. Piotrowska et al. [29] detected 7 MET amplifications in 32 patients with osimertinib resistance, accounting for approximately 22%. In the AURA3 study, MET amplification was seen in approximately 19% of the plasma samples from 73 patients taking antibiotics with the second-line treatment of osimertinib, exceeding the percentage of EGFR C797 secondary mutations (15%), which is the most common drug resistance mechanism. A similar analysis was performed in other third-generation EGFR-TKI studies. In a study of AC0010-resistant EGFR-mutant NSCLC patients, MET amplification as detected in only 1 of 16 patients, accounting for approximately 6.25% [30].

Currently, most studies have focused on the resistance mechanisms of osimertinib and other third-generation EGFR-TKI second-line treatments. There are few studies on the first-line treatment of EGFR mutation-positive advanced NSCLC with osimertinib, which may be related to its recent approval. A study presented at the 2018 European Society for Medical Oncology (ESMO) annual meeting used NGS technology to analyze 91 plasma samples from patients undergoing first-line treatment with osimertinib; MET amplification was detected in 15% of the samples and EGFR in 7% of the samples. The C797S mutation suggests that MET amplification is the most common acquired resistance mechanism [31].

## MET amplification leads to treatment strategies for EGFR-TKI resistance

**Fig. 2:**
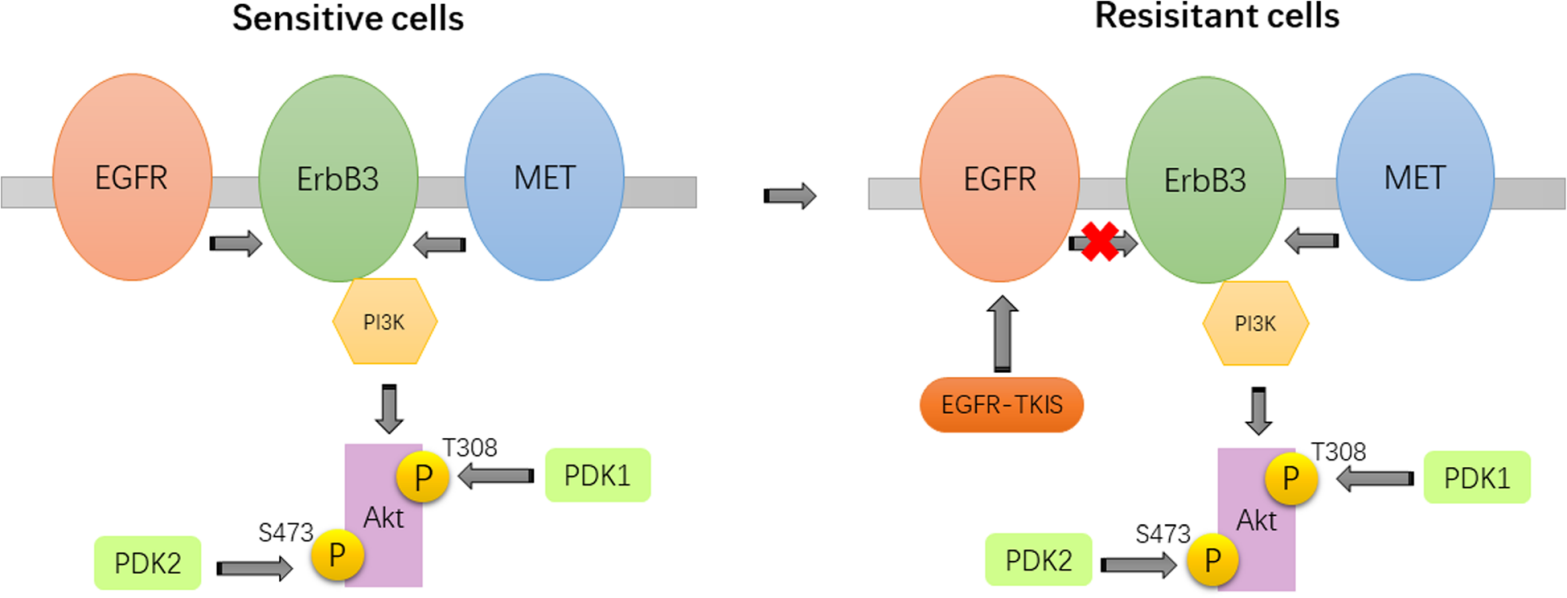
MET amplification causes EGFR-TKI resistance by activating EGFR-independent phosphorylation of ErbB3 and consequent downstream activation of the PI3K/AKT pathway, providing a resistance mechanism that can bypass the effects of an EGFR-TKI. MET can also activate PI3K/Akt signaling through ErbB3

The underlying mechanism by which MET amplification leads to EGFR-TKI resistance is associated with the activation of the EGFR-independent phosphorylation of ErbB3 and the downstream activation of the PI3K/AKT pathway, providing a bypass signaling pathway even in the presence of EGFR-TKIs [9]. Therefore, it is necessary to simultaneously inhibit EGFR and MET to overcome the EGFR-TKI resistance caused by MET amplification (Figs. 2, 9]. A number of studies have shown that HCC827/ER cells and HCC827/AR cells have MET amplification in vitro and in vivo, and gene-knockout MET or small molecule MET inhibitors combined with osimertinib can effectively inhibit the growth of these two cells [19, 21, 32, 33]. A similar finding was also reported in the clinic. In patients who were resistant to osimertinib and who were tested for MET expansion, a combination of EGFR-TKI and crizotinib was used, and the effect was evaluated as partial response (PR) [11]. In another case, a patient with NSCLC carrying the EGFR L858R mutation was treated with erlotinib in the first line. After progression, MET amplification was found in the genetic test. The combination of full-dose osimertinib and crizotinib was given. The therapeutic effect was evaluated as PR. The combination was well tolerated in patients [34]. This finding suggests that MET inhibitors combined with osimertinib or other third-generation EGFR-TKIs for EGFR-TKI resistance induced by MET amplification may be a new therapeutic strategy that needs further validation in the clinic.

In addition, the inhibition of MEK by small molecule MEK inhibitors such as trametinib (GSK1120212) is also an effective and feasible solution to overcome MET-induced acquired resistance to osimertinib [35]. Studies have shown that different MEK inhibitors can inhibit the growth of HCC827/AR cells or tumor growth in vitro when combined with osimertinib and can induce apoptosis [35]. Compared with MET inhibitors, MEK inhibitors not only inhibit the growth of cells with MET amplifications but also inhibit the growth of other drug-resistant cell lines with different potential mechanisms [35]. Thus, the use of MEK inhibitors is also an option in clinical treatment.

Currently, two small molecule c-MET inhibitors, cabozantinib and crizotinib, have been approved by regulatory authorities for the treatment of selected cancer types, but several novel c-MET inhibitors are currently in trials for different settings [34, 36–38]. MET inhibitors can be divided into three categories: small molecule MET receptor inhibitors (such as crizotinib, tivantinib, savolitinib, tepotinib, cabozantinib and foretinib), monoclonal antibodies targeting the MET receptor (such as onartuzumab) and anti-HGF antibodies (such as ficlatuzumab and rilotumumab) [17, 39–44]. Some MET inhibitors in combination with EGFR-TKIs have been studied in patients, but this treatment regimen has not shown significant efficacy in patients with unselected NSCLC. Some positive results were obtained in patients with MET amplification or overexpression. Therefore, MET may be an effective therapeutic target for NSCLC patients with EGFR-TKI resistance and MET expansion [45].

Immunotherapy has emerged as a new topic in advanced lung cancer research and has made breakthroughs in the first-line and second-line treatment of advanced NSCLC [46–49]. However, immunotherapy is not effective in patients with EGFR-mutant NSCLC [49]. Recent studies have shown that in MET-amplified tumors, treatment with MET inhibitors can counteract interferon-gamma-mediated induction of the PD-1 ligand [50]. In studies of liver cancer cell lines and mice with orthotopic tumors, MET mediated the phosphorylation of and activated GSK3B, leading to decreased PDL1 expression. When combined with a MET inhibitor, anti-PD1 and anti-PDL1 antibodies elicited an additive effect to slow the growth of HCC cells in mice [51]. Therefore, whether an anti-MET drug can be combined with a PD-1 or PD-L1 inhibitor to treat EGFR-mutant NSCLC resistant to osimertinib due to MET amplification and/or overactivation requires further investigation.

## Conclusions

A significant correlation between MET receptor overexpression/hyperactivation and poor outcomes has been demonstrated in different solid tumors, including NSCLC. Many new molecules that either target MET or act as multitarget inhibitors are emerging and exhibit antitumor activity. MET inhibitors have shown promising antitumor activities in preclinical and early phase clinical trials of several tumor types, although the results of most phase III trials with these agents have been less encouraging. But with the efforts of translational and clinical research, an increasing number of MET-targeted therapies will surely have a positive impact on lung cancer outcomes, and these treatments should be included as one of the possible therapeutic options for patients with NSCLC.

Osimertinib is currently approved by the FDA for the treatment of EGFR-mutant NSCLC with a T790 mutation after relapse following first- or second-generation EGFR-TKI therapy (second-line). However, approximately 20% of these patients are not sensitive to treatment with osimertinib. Based on our findings, we believe that it is necessary to detect the MET status prior to osimertinib treatment. Patients with EGFR-mutant NSCLC may be insensitive to osimertinib or other third-generation EGFR-TKIs. Combination therapy with a combination of MET or MEK inhibitors may be considered for these patients. In the last few years, anti-PD1/PD-L1 drugs have become a new paradigm in oncology, and based on current research, anti-PD1 and anti-PDL1 antibodies combined with a MET inhibitor may finally represent an effective treatment of EGFR-mutant NSCLC.

MET amplification and MET protein expression are often detected in clinical practice, but there are few studies on p-MET. MET-amplified EGFR-mutant NSCLC cell lines not only have high levels of MET but also have high levels of p-MET [19]. Therefore, the detection of p-MET and its potential as a predictive marker should be explored.

## Data Availability

Not applicable as no datasets were generated or analyzed.

## Abbreviations

CNV: Copy number variation
ddPCR: Droplet digital PCR
EGFR-TKIs: EGFR-tyrosine kinase inhibitors
ESMO: European society for medical oncology
FISH: Fluorescence in situ hybridization
HGF: Hepatocyte growth factor
IHC: Immunohistochemistry
NGS: Next-generation sequencing.
NSCLC: Non-small-cell lung cancer
PR: Partial response
